# Editorial: Women in oral health promotion: 2021

**DOI:** 10.3389/froh.2022.1023586

**Published:** 2022-09-27

**Authors:** Marília Goettems

**Affiliations:** Department of Social and Preventive Dentistry, School of Dentistry, Federal University of Pelotas, Pelotas, Brazil

**Keywords:** oral health promotion, access to oral health care, disparities in oral health, oral health of special populations, lifestyle factors

**Editorial on the Research Topic** Women in oral health promotion: 2021

Discrimination is a serious issue in the scientific world and in nearly all countries men dominate scientific production. In dentistry, women remain underrepresented in academia, and this gap is widened whenever each career step is progressed (1). Whether consciously or not, gender bias is a severe problem that affects women’s careers at different levels and can be decisive in academic success (2). Increased efforts to combat subtler forms of gender bias in scholarly publishing are needed.

Therefore, this research topic was intended to promote the work of women researchers, across all fields of Oral Health Promotion. Four high-quality articles were published, whose first or last authors are a researcher who identifies as a woman. All studies are original research, and they were conducted by well-known researchers in Chile and in the United States (US).

Morales et al. investigated the impact of periodontal therapy in terms of oral health-related quality of life (OHRQoL) during the COVID-19 pandemic in a cohort of diabetic patients with stage III-IV periodontitis. Periodontal clinical parameters including clinical attachment loss (CAL), probing pocket depth (PPD) and bleeding on probing (BOP) as well as glycated hemoglobin (HbA1c) were evaluated at baseline and 3 months follow-up prior the pandemic. The OHRQoL changes by means of Oral Health Impact Profile (OHIP-14) and a self-reported oral health questionnaire were assessed at baseline (prior pandemic) and during the pandemic *via* telemonitoring. The findings revealed an increase in the OHRQoL between baseline and the COVID confinement, indicated by the overall improvement of the OHIP-14 scores. Thus, periodontal therapy tends to improve the oral health-related quality of life, despite the confinement due to the COVID-19 pandemic.

Hammersmith et al. evaluated the feasibility, acceptability, and sustainability of a pilot teledentistry program in a children's hospital network in Ohio. The study described how teledentistry can be used to facilitate consultation and communication between dentists and medical personnel and revealed barriers in connecting the two. Although the pilot utilization of teledentistry technology was extremely low, medical personnel’s experiences were generally positive when teledentistry was used. Specifically, medical personnel largely used teledentistry for patients with dental trauma.

Dennison and Seward discuss potential solutions to address oral health care challenges in Oklahoma (US). According to the authors, Oklahoma’s oral health statistics reflect a population with high rates of childhood tooth decay, adult tooth loss and absence of regular oral health appointments primarily due to cost. Deficiencies are discussed and strategies are outlined, including efforts at all levels of government, innovations of health care delivery and recognition of the unique needs of Oklahoma American Indian population.

Donald et al. investigated dental needs among mid-to-older deaf and hard of hearing (DHH) women and identified social determinants of health that may place them at higher risk for unmet dental health needs. Data drawn from Communication Health domain in the PROMIS-DHH Profile and oral health data from the National Health and Nutrition Examination Survey were used. Despite the significant number of DHH people living in the U.S., oral health research on deaf and hard of hearing people who specifically use American Sign Language (ASL) is virtually non-existent. Based on their results, authors suggest actions toward reducing oral health disparities for DHH patients with less years of education or low oral health literacy, including allowing as many questions as possible to be asked and taking time to educate DHH patients.

At present, less than 30% of researchers worldwide are women. Long-standing biases and gender stereotypes are discouraging girls and women away from science-related fields. Science and gender equality are, however, essential to ensure sustainable development as highlighted by UNESCO. In order to change traditional mindsets, gender equality must be promoted, stereotypes defeated, and girls and women should be equally represented in the proportion of researchers worldwide. The work presented here highlights the diversity of research performed by women across the entire breadth of Oral Health Promotion research and presents advances in theory, experiment, and methodology with applications to compelling problems.
